# Orexinergic Input to Dopaminergic Neurons of the Human Ventral Tegmental Area

**DOI:** 10.1371/journal.pone.0083029

**Published:** 2013-12-20

**Authors:** Erik Hrabovszky, Csilla S. Molnár, Beáta Á. Borsay, Péter Gergely, László Herczeg, Zsolt Liposits

**Affiliations:** 1 Laboratory of Endocrine Neurobiology, Institute of Experimental Medicine, Hungarian Academy of Sciences, Budapest, Hungary; 2 Department of Forensic Medicine, Faculty of Medicine of the University of Debrecen, Debrecen, Hungary; 3 Department of Neuroscience, Faculty of Information Technology, Pázmány Péter Catholic University, Budapest, Hungary; Kaohsiung Chang Gung Memorial Hospital, Taiwan

## Abstract

The mesolimbic reward pathway arising from dopaminergic (DA) neurons of the ventral tegmental area (VTA) has been strongly implicated in reward processing and drug abuse. In rodents, behaviors associated with this projection are profoundly influenced by an orexinergic input from the lateral hypothalamus to the VTA. Because the existence and significance of an analogous orexigenic regulatory mechanism acting in the human VTA have been elusive, here we addressed the possibility that orexinergic neurons provide direct input to DA neurons of the human VTA. Dual-label immunohistochemistry was used and orexinergic projections to the VTA and to DA neurons of the neighboring substantia nigra (SN) were analyzed comparatively in adult male humans and rats. Orexin B-immunoreactive (IR) axons apposed to tyrosine hydroxylase (TH)-IR DA and to non-DA neurons were scarce in the VTA and SN of both species. In the VTA, 15.0±2.8% of TH-IR perikarya in humans and 3.2±0.3% in rats received orexin B-IR afferent contacts. On average, 0.24±0.05 and 0.05±0.005 orexinergic appositions per TH-IR perikaryon were detected in humans and rats, respectively. The majority (86–88%) of randomly encountered orexinergic contacts targeted the dendritic compartment of DA neurons. Finally, DA neurons of the SN also received orexinergic innervation in both species. Based on the observation of five times heavier orexinergic input to TH-IR neurons of the human, compared with the rat, VTA, we propose that orexinergic mechanism acting in the VTA may play just as important roles in reward processing and drug abuse in humans, as already established well in rodents.

## Introduction

The neuropeptides orexin A and orexin B (also known as hypocretin 1 and hypocretin 2) have been implicated in a variety of behavioral states including feeding [1], sleep and arousal [2], reward processing and drug abuse [3]. As reviewed recently, orexinergic signaling modulates many responses to drugs of abuse and food, such as hyperlocomotor activity and sensitization, drug withdrawal, self-administration and conditioned place preference [4].

In rodents, the perikarya of orexin synthesizing neurons are located in the dorsal medial hypothalamus, perifornical area and the lateral hypothalamus [1], [5]–[7]. Their axonal projections to the midbrain ventral tegmental area (VTA) [7]–[9] have been implicated in the wide effects of orexins on the mesolimbic reward pathway [7], [9], [10]. Both dopaminergic (DA) and GABAergic neurons of the VTA receive afferent contacts from orexinergic axons [9]–[11]. Because synapses are rarely detectable in these juxtapositions, orexins have been proposed to act mainly via non-synaptic mechanism upon the A10 DA neurons and GABAergic interneurons of the VTA [11].

The rodent VTA contains both orexin receptor forms (Ox1R and Ox2R) [12]–[14], with particularly high levels of Ox2R [13]. The immunohistochemical detection of Ox1R and Ox2R in DA [12], [14] and non-DA [14] neurons indicates that both cell types of the VTA represent direct targets for orexinergic actions. In accordance with these neuroanatomical observations, electrophysiological evidence indicates that orexins can activate both DA and non-DA cells of the VTA via direct postsynaptic mechanisms [14]. Furthermore, ventricular infusion of orexin A induces c-Fos expression in distinct subsets of VTA DA neurons [15] and orexin administration into the VTA increases dopamine efflux in the terminal fields of the mesolimbic reward pathway, the medial prefrontal cortex [16] and the nucleus accumbens [12]. Acute application of orexin A into the VTA potentiates N-methyl-D-aspartate receptors of local neurons and facilitates the plasticity induced by drugs of abuse [17], [18]. Orexinergic mechanisms play critical roles in the rewarding effect of morphine through the activation of the mesolimbic DA pathway. Accordingly, morphine-induced place preference and hyperlocomotion observed in wild-type mice are absent in the prepro-orexin knockout mice [12]. Orexins appear to exert these behavioral effects mainly via acting in the VTA because intra-VTA injection of the selective Ox1R antagonist SB-334867A [1-(2-methylbenzoxazol-6-yl)-3-[1.5]naphthyridin-4-yl urea] significantly suppresses the morphine-induced place preference in rats [12].

Although the critical role of a direct orexinergic input to the VTA in reward processing and drug abuse has been well established in laboratory rodents [3], the functional significance of an analogous orexinergic projection in the human has not been explored. Therefore, in the present study we addressed the issue of whether orexinergic neurons provide a similar direct input to VTA DA neurons in the human as reported previously in the rat. To demonstrate this projection, we first carried out the dual-label immunohistochemical studies of tissue sections from *post-mortem* human midbrain samples and analyzed orexin B-immunoreactive (IR) neuronal contacts onto DA and non-DA neurons of the VTA. The surrounding substantia nigra (SN; pars compacta) was also studied. Notably, the firing of DA neurons at this site of the rat was unaffected by orexins, whereas orexins excited GABAergic neurons in the pars reticulata of the SN [19]. To explore neuroanatomical similarities/differences between the rat and the human, we next compared quantitatively the incidences of orexin B-IR axo-somatic contacts onto the VTA and SN DA neurons in the two species. Finally, the relative contributions of axo-somatic and axo-dendritic contacts to this communication were determined quantitatively in each midbrain region and species. Based on the similar neuroanatomical features of these orexinergic pathways in the two species, with about five times higher relative incidences of orexinergic contacts on individual VTA DA neurons in humans than in rats, we propose that orexin actions in the VTA may be critically involved in reward processing and drug addiction in the human, as it has been established well in rodents.

## Materials and Methods

### Ethics Statement

Human brain tissue samples were obtained at autopsy from the Forensic Medicine Department of the University of Debrecen, with the permission of the Regional Committee of Science and Research Ethics (DEOEC RKEB/IKEB: 3183-2010) and according to the Hungarian Law (1997 CLIV and 18/1998/XII.27. EÜM Decree/). All personal data were anonymized.

Experiments on rats were carried out in accordance with the Council Directive of 24 November 1986 of the European Communities (86/609/EEC) and were reviewed and approved by the Animal Welfare Committee of the Institute of Experimental Medicine (No. A5769-01).

### Human Subjects

Human VTA and SN tissue samples were used from five male subjects (Ages: 37, 40, 50, 57 and 59 years). Autopsies were carried out within 48 h after death (four by suicidal hanging and one by hypothermia). Known patient histories did not include preexisting neurological or endocrine disorders and information about potential drug abuse was not available.

### Animals

Five adult (250–350 g body weight) male Wistar rats (Charles River, Germany) were used for the comparative analysis between the rat and the human of the orexinergic inputs to midbrain DA neurons. The rats were housed in a light- (12-h light, 12-h dark cycle, lights on at 0700 h) and temperature-controlled (22±2 C) environment, with free access to standard food and tap water.

### Tissue Preparation for Immunohistochemistry

#### Preparation of human tissue sections

Human tissue blocks containing the VTA were dissected out and cut in half in the midsagittal plane. The blocks were trimmed further so that coronal sections to be cut could later be accommodated on regular microscope slides. The blocks were rinsed with running tap water and then, immersed into 4% formaldehyde in 0.1 M phosphate buffer saline (PBS; pH 7.4) for 7–14 days at 4°C. The fixed tissues were infiltrated with 20% sucrose for 5 days at 4°C. The right halves were placed in freezing molds, surrounded with Jung tissue freezing medium (Leica Microsystems, Nussloch Gmbh, Germany; diluted 1∶1 with 0.9% sodium chloride solution), snap-frozen on powdered dry ice, and sectioned coronally at 30 µm with a Leica SM 2000 R freezing microtome (Leica Microsystems). Sections 14.6–25.2 mm behind the anterior commissure corresponding to atlas plates 33–41 of the human brain atlas of Mai [20] were collected and stored permanently in anti-freeze solution (30% ethylene glycol; 25% glycerol; 0.05 M phosphate buffer; pH 7.4) at −20°C before use in immunohistochemical studies.

#### Preparation of rat tissue sections

The rats were anesthetized with a cocktail of ketamine (25 mg/kg), xylavet (5 mg/kg), and pipolphen (2.5 mg/kg) in saline and then, perfused transcardially with 150 ml fixative solution containing 4% formaldehyde (Sigma Chemical Co., St. Louis, MO) in 0.1 M PBS (pH 7.4). The hypothalami were dissected and soaked in 25% sucrose overnight for cryoprotection. Then, 30-µm-thick free-floating coronal sections 5.20–5.80 mm posterior to Bregma were cut from the VTA according to atlas plates 40–43 of Paxinos and Watson [21] with a freezing microtome and stored in cryoprotectant.

### Tissue Pretreatments for Immunohistochemistry

Prior to immunohistochemistry, human and rat sections were rinsed abundantly in PBS and pretreated with a mixture of 0.5% H_2_O_2_ and 0.2% Triton X-100 for 30 min. In addition, human sections underwent antigen retrieval using 0.1 M citrate buffer (pH = 6.0) at 80°C for 30 min [22].

### Dual-immunoperoxidase Detection of Orexin B-IR Inputs to Tyrosine Hydroxylase Synthesizing Neurons

Every 24th section of the VTA from each human individual and every 4th section from each rat was incubated in a goat polyclonal orexin B antiserum (sc-8071; C-19, 1∶50,000; Santa Cruz Biotech Inc., Santa Cruz, CA) for 48 h at 4°C. This well-characterized antiserum [23], [24] labels orexinergic neurons in various species and provides no immunostaining of hypothalamic tissues from orexin deficient mice [24]. Human hypothalamic orexinergic neurons in our tissue samples showed the same distribution using this antiserum as described by others using other antibodies [25]. The primary antiserum was reacted with biotinylated secondary antibodies (donkey biotin-SP-anti-goat IgG; Jackson ImmunoResearch Laboratories, West Grove, PA, USA; 1∶500) and the ABC Elite reagent (Vector, Burlingame, CA; 1∶1000) for 60 min each. The peroxidase signal was visualized with nickel-intensified diaminobenzidine chromogen and then, post-intensified with silver-gold [26]. Next, TH neurons were detected with chicken TH antibodies from AVES laboratories (Aves Laboratories Inc., Tigard, OR; #TYH; 1∶300, 48 h). The primary antibodies were reacted with donkey biotin-SP-anti-chicken IgY (Jackson ImmunoResearch; 1∶500; 60 min) and the ABC Elite reagent (1∶1000; 60 min) and then, the peroxidase signal was developed using brown diaminobenzidine. The signal pattern provided by these antibodies agreed with the known distribution of DA neurons in the pars compacta of the SN and in other brain regions.

### Section Mounting and Coverslipping

The immunostained sections were mounted onto Silanized microscope slides from Elvanol and dried. Some were stained with cresyl violet in order to also visualize non-DA cell bodies of the VTA. Finally, all sections were dehydrated with 95% (5 min), followed by 100% (2×5 min) ethanol, cleared with xylene (2×5 min) and coverslipped with DPX mounting medium (Sigma, St. Louis, USA). Representative light microscopic images were prepared with an AxioCam MRc 5 digital camera mounted on a Zeiss AxioImager M1 microscope and using the AxioVision 4.6 software (Carl Zeiss, Göttingen, Germany).

### Quantitative Light Microscopic Analysis

To address species similarities/differences in the orexin B-IR input to TH-IR neurons of the VTA and SN, several immunohistochemical parameters of these connections were analyzed quantitatively and data obtained from 5 rats and 5 humans compared. In each of these comparative studies the counting of neuronal appositions was carried out using a X63 oil-immersion objective lense to determine the number of axonal contacts along the outlines of TH-IR elements, using consistently applied stringent criteria for contacts [27], [28]. In brief, the orexinergic axon and the TH-IR profile had to be in the same focus plane without any visible intervening gap and instances of partial overlap were not considered. The analysis of axosomatic contacts included 1213 DA neurons from the human VTA (28 half sections; 5 individuals), 3860 DA neurons from the human SN (5 sections; 5 individuals), 2729 DA neurons from the rat VTA (5 sections; 5 rats) and 2232 DA neurons from the rat SN (5 sections; 5 rats). First, the percentages of TH-IR perikarya that received at least one afferent contact were determined separately in the VTA and the pars compacta of the SN in both humans and rats. Then, the average incidence of contacts per TH-IR soma was determined in each region and species. The above parameters of the two species were compared with Student’s t-test for independent samples (Statistica 11 software package; StatSoft Inc., Hungary). Species differences were considered significant at p<0.05. Finally, the first randomly encountered one hundred orexin B/TH appositions were analyzed in one section of each human subject and each rat to determine the percent distribution of orexin B-IR inputs on the dendritic (including shafts and dendritic branches) and the somatic compartments of DA neurons.

## Results

### Distribution of TH-IR DA Neurons in the VTA and the SN

**Figure 1:**
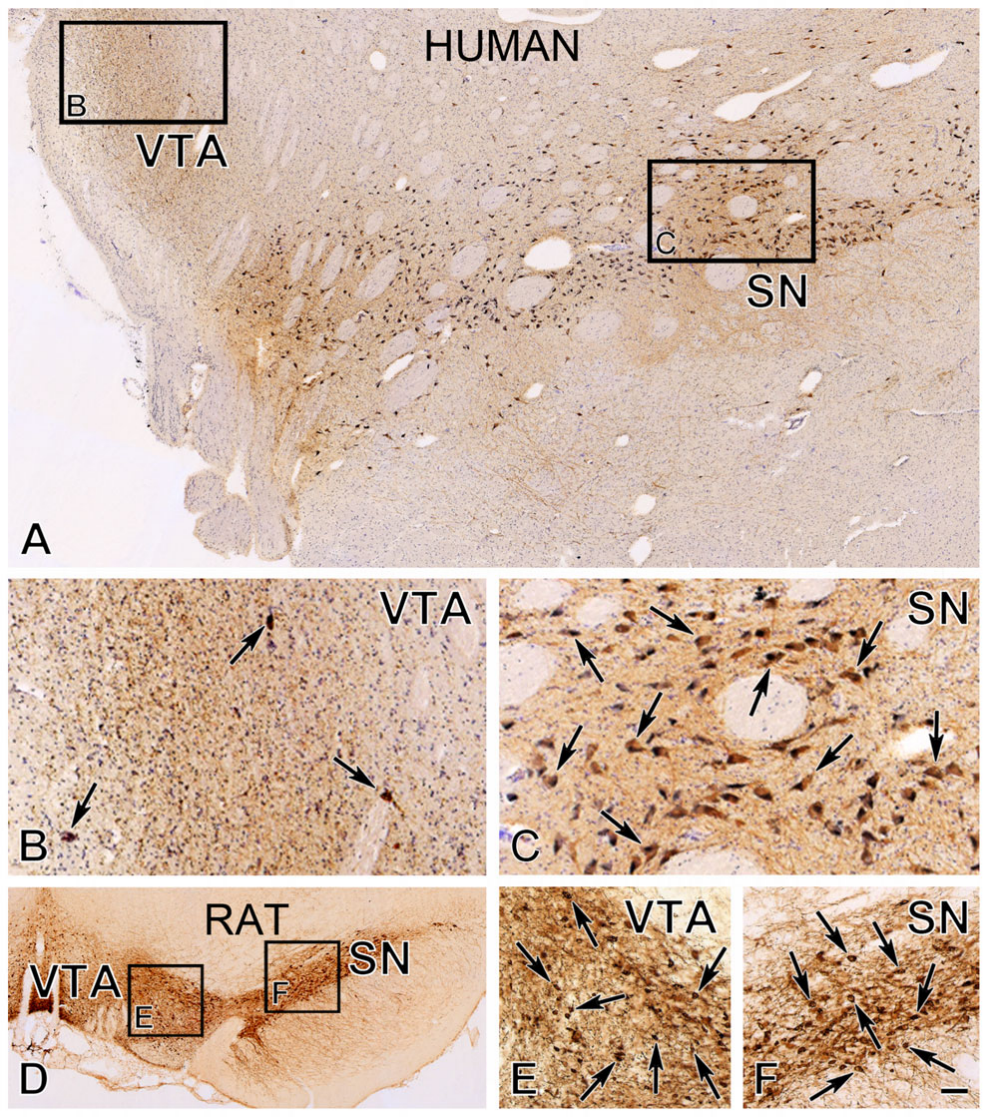
Immunohistochemical detection of dopaminergic neurons in the VTA and the SN of the human and the rat. Representative low-power images of immunostained sections from an adult male human (**A**) and rat (**D**) illustrate the distribution of tyrosine hydroxylase (TH)-immunoreactive (IR) dopaminergic neurons in the ventral tegmental area (VTA) and pars compacta of the substantia nigra (SN). Medium-power images (insets **B**, **C**, **E** and **F**) reveal that the pars compacta of the SN contains densely-packed dopaminergic neurons (arrows) in both species. In contrast, while dopaminergic neurons are distributed loosely in the human VTA (arrows in **B**), they exhibit a relatively high regional cell density in the VTA of the rat (arrows in **E**). Cresyl violet staining in **A–C** visualizes non-dopaminergic perikarya. Scale bar = 200 µm in **A**, **D** and 66 µm in **B, C, E, F**.

**Figure 2:**
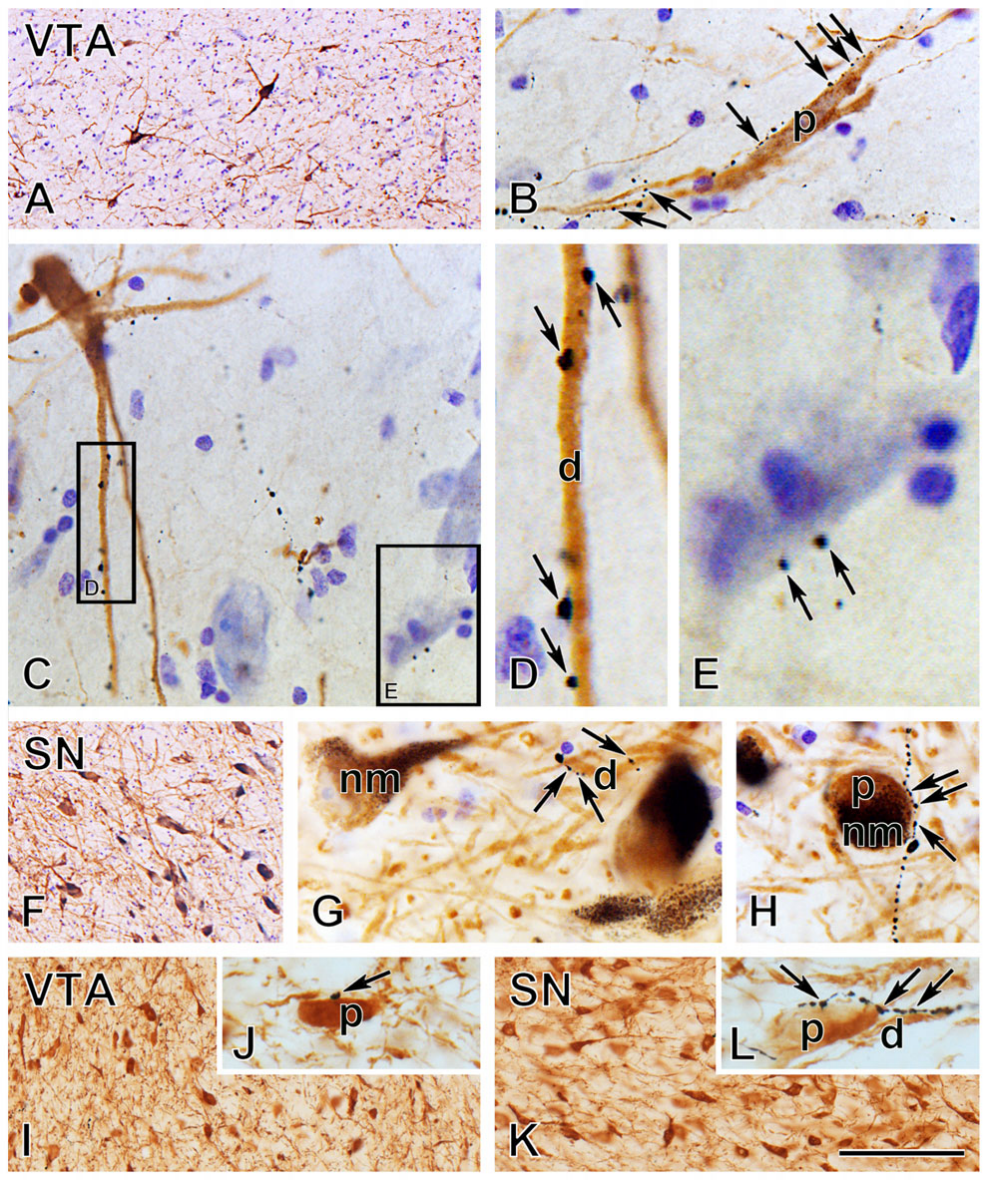
Orexin B-immunoreactive inputs to dopaminergic and non-dopaminergic neurons in the human and rat VTA and SN. Images of histological samples from an adult male human (**A–H**) and rat (**I–L**) illustrate orexin B (black) and tyrosine hydroxylase (TH; brown) immunoreactivities visualized with the silver-gold intensified nickel-diaminobenzidine and diaminobenzidine chromogens, respectively, in the ventral tegmental area (VTA; **A–E**, **I**, **J**) and the pars compacta of the substantia nigra (SN; **F–H**, **K**, **L**). Cresyl violet was applied in **A–H** to also reveal non-dopaminergic perikarya in human midbrain sections. Arrows in high power photomicrographs point to the sporadically encountered neuronal appositions between orexin B-immunoreactive (IR) axons and TH-IR (**B–D, G, H, J, L**) or TH-immunonegative (**E**) perikarya and dendrites. Dopaminergic neurons of the human VTA (**A–E**) form a loose cell mass in which TH-IR dopaminergic and Nissl-labeled non-dopaminergic neurons intermingle. Orexin B-IR appositions can only be observed on a relatively small subset of the dopaminergic cell bodies (**B**) and dendrites (**B, D**). Framed regions in **C** are shown in high-power micrographs **D** and **E** and illustrate orexinergic contacts on a TH-IR dendrite (**D**) and a TH-immunonegative Nissl-stained perikaryon (**E**), respectively. The pars compacta of the human SN exhibits a high density of dopaminergic cell bodies which contain dark brown neuromelanin (nm) granules (**F–H**). High-power images illustrate the infrequent apposition of orexin B-IR axons to the dendrites (**G**) and perikarya (**H**) of a small subset of TH-IR dopaminergic neurons. The VTA of the rat (**I**) exhibits a higher density of dopaminergic neurons, compared with the human VTA (**A**). Orexinergic contacts (**J**) on these neurons are rare. Similarly to the human, dopaminergic neurons of the rat form a compact cell population in the SN (**K**) and receive orexin B-immunoreactive inputs infrequently (**L**). Comparison of the above innervation patterns in the two species provides quantitative evidence for significantly heavier input frequencies in the human (Figs. 3 and 4), whereas Fig. 5 illustrates that the vast majority of orexinergic inputs target the dendritic compartment of dopaminergic neurons in both regions of both species. p, TH-IR perikarya; d, TH-IR dendrites; nm, dopaminergic cell bodies containing high levels of neuromelanin pigment. Scale bar = 10 µm in **D**, **E**, **G**, **H**, 30 µm in **B**, **C**, **J**, **L** and 130 µm in **A**, **F**, **I**, **K**.

The light microscopic analysis of immunostained sections from adult male humans and rats revealed similarities as well as differences in the distribution of TH-IR DA neurons in the VTA and the pars compacta of the SN (Figs. 1 and 2).

In humans, DA neurons of the VTA formed a loose cell population intermingling with TH-immunonegative non-DA neurons that were only Nissl-stained (Figs. 1B, 2A–E). The DA neurons showed variable morphology and contained no or only low amounts of neuromelanin pigment (Figs. 2A–D). The DA neurons in the pars compacta of the SN, in turn, formed a dense cell mass and their cytoplasm enclosed numerous golden-brown neuromelanin granules (Figs. 1C, 2F–H).

In rats, DA cell bodies occurred at high densities both in the VTA (Figs. 1E, 2I, J) and the SN (Figs. 1F, 2K, L).

### Orexin B-IR Innervation of TH-IR Neurons in the VTA and SN

In humans, scattered orexin B-IR axons, labeled with black silver-gold-intensified Ni-DAB, were observed in the VTA (Figs. 2B–E) as well as the SN (Figs. 2G, H). In both regions, orexinergic axons established axo-dendritic (Figs. 2B–D, G) and, more sporadically, also axo-somatic (Figs. 2B and H) appositions onto DA neurons. The majority of TH-IR cell bodies and dendrites were not surrounded and contacted by orexinergic axons (not shown). The rarely encountered axo-somatic contacts also targeted TH-immunonegative non-DA neurons, as evidenced in Nissl-stained preparations (Fig. 2E).

In rats, orexin B-IR axons provided even more scarce inputs to DA neurons as in humans, both in the VTA (Figs. 2I, J) and the SN (Figs. 2K, L).

### Results of Quantitative Comparisons between Species

Quantitative comparisons between species revealed the following similarities/differences in the innervation patterns of DA neurons.

**Figure 3:**
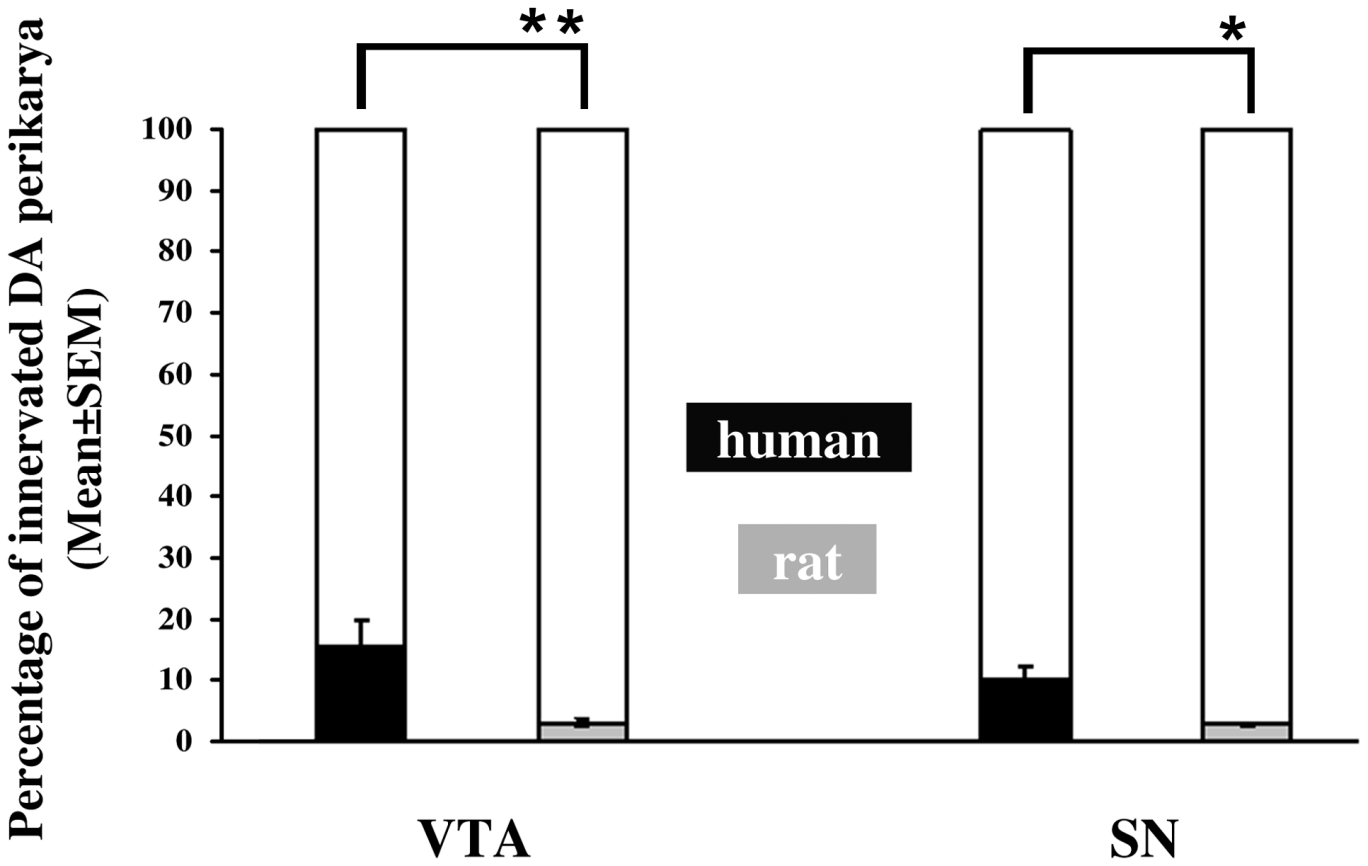
Percentages of dopaminergic somata receiving orexinergic innervation in the VTA and the SN. Bar graph illustrates the low percentages of tyrosine hydroxylase-immunoreactive dopaminergic neuronal cell bodies that receive innervation from orexin B-immunoreactive axons in the ventral tegmental area (VTA) and substantia nigra (SN) of adult male humans and rats. Note that while the axo-somatic innervation is quite sparse in both species, the percentage of dopaminergic cell bodies receiving orexinergic input is 5-times higher in the human compared with the rat VTA and 2.2-times higher in the human compared with the rat SN. *p<0.05; **p<0.01.

In the VTA, the incidences of TH-IR cell bodies receiving orexin B-IR contacts were low in the human (15.0±2.8%) and even lower (3.2±0.3%) in the rat. Similarly in the SN, only 8.7±1.6% of DA somata in the human and 4.0±0.9% in the rat received orexin B-IR appositions (Fig. 3). Species differences were statistically significant by t-test (N = 5, t = 4.17, p = 0.003 for the VTA and N = 5, t = 2.6, p = 0.031 for the SN).

**Figure 4:**
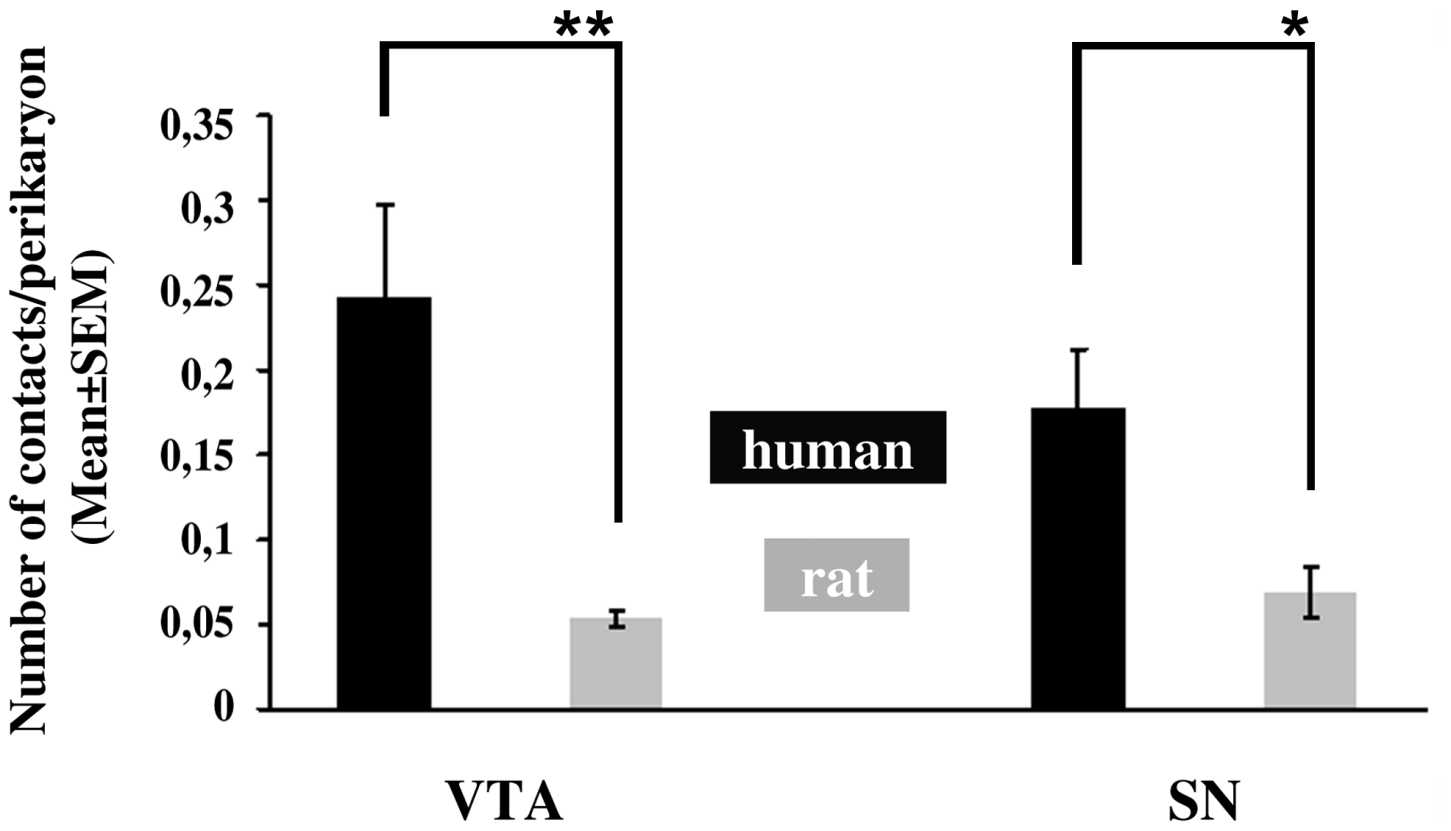
Mean incidences of orexin B-immunoreactive afferent contacts onto individual dopaminergic cell bodies. The mean incidences of orexin B-immunoreactive afferent contacts onto the cell bodies of dopaminergic neurons in the ventral tegmental area (VTA) and the substantia nigra (SN) are low both in adult male humans and rats. Individual dopaminergic cell bodies receive 5-times more orexinergic appositions in the human compared with the rat VTA and 2.6-times more appositions in the human compared with the rat SN. *p<0.05; **p<0.01.

In the VTA, the mean frequency of axosomatic appositions (contacts/TH-IR perikaryon) was 0.24±0.05 in the human and only 0.05±0.005 in the rat. In the SN, the mean incidence of these axosomatic contacts was 0.18±0.03 in the human and 0.07±0.01 in the rat (Fig. 4). Species differences were statistically significant by t-test (N = 5, t = 3.52, p = 0.008 for the VTA and N = 5, t = 2.87, p = 0.021 for the SN). Overall, in the VTA as well as the SN, both the incidence of TH-IR cell bodies receiving orexin B-IR input and the number of orexinergic appositions per TH-IR perikaryon were significantly higher in humans than in rats.

**Figure 5:**
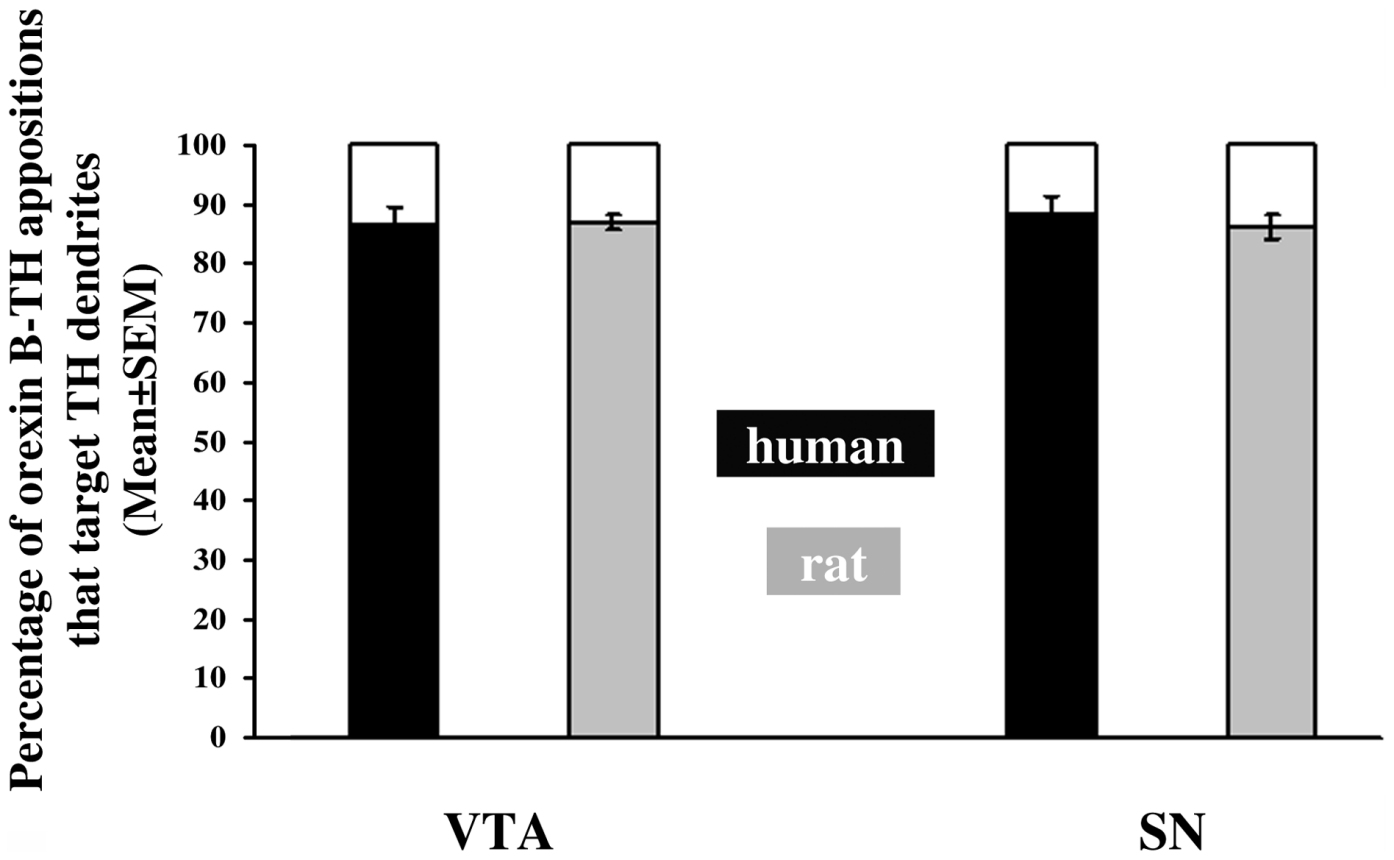
Percentages of orexin B-immunoreactive contacts targeting the dendritic compartment of dopaminergic neurons. The mean percentages of orexin B-immunoreactive contacts that target the dendritic compartment of dopaminergic neurons were calculated from the first randomly encountered one hundred orexin B/TH appositions in each human individual and in each rat. Both in the ventral tegmental area (VTA) and the substantia nigra (SN) and both in humans and rats, the vast majority (86–88%) of orexinergic inputs to dopaminergic neurons is axo-dendritic.

Finally, humans and rats showed a similarity in the preferential targeting of the orexinergic inputs to the dendritic *vs.* the somatic compartment of DA neurons at both anatomical sites. 86.6±2.7% of the orexin B-IR apposition in the human and 87.0±1.3% in the rat VTA were directed to the dendritic compartment of DA neurons (Fig. 5). Similarly in the SN, 88.4±2.9% from the first 100 randomly encountered orexin B/TH appositions in each human subject and 86.2±2.1% in each rat targeted dendrites.

## Discussion

This immunohistochemical study provides evidence for direct orexinergic inputs to TH-IR DA neurons of the human VTA and SN. The patterns of orexinergic innervation in the human were similar to the analoguous orexinergic pathways in the rat, except for three aspects. First, the regional density of TH-IR cell bodies detectable in the VTA of the human was much lower than in the rat, resulting in lower numbers of DA cells analyzed in the human VTA. Second, the percentages of DA neurons (both in the VTA and the SN) innervated by orexinergic axons were several times higher in the human, compared with the rat. Third, the incidence of orexinergic inputs to individual DA neurons was also several times higher in the human compared with the rat. In both species and both regions, the majority (86–88%) of orexinergic inputs targeted the dendritic compartment of DA neurons.

Orexins originally described in 1998 by two independent groups [1], [6] are synthesized in the dorsal medial hypothalamus, perifornical area and the lateral hypothalamus [1], [5]–[7], [25] and send extensive projections to the central nervous system [7]–[9].

The wide effects of orexins on the mesolimbic reward pathway of laboratory rodents have been established in a number of behavior tests. At least some drugs of abuse can act on orexinergic neurons directly while eliciting these behavioral responses. Orexinergic neurons contain µ opioid receptor [32] which may underlie the mechanism whereby morphine withdrawal by naloxone- or naltrexone following chronic morphine treatment, induces cAMP response element (CRE)-mediated transcription in CRE-LacZ reporter mice as well as c-Fos and orexin expression in orexinergic neurons of the lateral hypothalamus [32]. The somatic responses to withdrawal are attenuated in the presence of the Ox1R antagonist SB-334867 [33] and in orexin knock-out mice [32]. In self-administration paradigms, orexin receptor antagonists are capable of inhibiting the self-administration of nicotine [34] and alcohol [35], [36]. Orexinergic neurons are also involved in the reinstatement of extinguished responses to drugs of abuse. Accordingly, stimulation of lateral hypothalamic orexinergic neurons, or microinjection of orexin A into the VTA, can reinstate an extinguished morphine place preference [30]. Furthermore, intracerebroventricular administration of orexin A is capable of reinstating extinguished responses in animals trained to self-administer cocaine and food reinforcers [37] and similarly, extinguished cocaine seeking behavior can be reinstated by intra-VTA administration of orexin A [38]. Stimuli linked to ethanol availability can also increase Fos expression in orexin neurons, as shown in a reinstatement model of relapse [39]. Reinstatement of extinguished alcohol self-administration by alcohol-associated cues is absent in rats treated with SB-334867 [35]. The above and other regulatory effects of orexinergic neurotransmission on the mesolimbic reward pathway have been summarized in a number of recent review articles 4,29,30,40,41.

The relevance of animal data to the putative orexinergic regulation of the human reward circuitry has been unclear. In the present study we first established that a direct orexinergic projection to VTA DA neurons also exists in the human. Next, we compared several quantitative aspects of this communication pathway between the human and the rat species. First, we found that the input was not abundant in either the human or the rat and only low subsets of VTA DA perikarya were contacted by orexinergic axons; the percentage of DA neurons innervated by orexin B-IR fibers was five times higher in the human (15.0±2.8%) than in the rat (3.2±0.3%). Second, the mean incidence of axosomatic contacts was also found to be low in both species, but again, about five times higher in the human (0.24±0.05) than in the rat (0.05±0.005). Finally, in both species about 87% of the orexinergic contacts were observed on the dendritic compartment of VTA DA neurons. Based on the observations that i) the percentage of VTA DA neurons receiving orexinergic innervation ii) and the number of orexinergic contacts on individual VTA DA neurons were five times higher in the human than in the rat, we propose that orexinergic mechanisms acting in the human VTA play critical roles in reward processing and drug abuse, as already established well in laboratory rodents [4], [29]–[31].

While our immunohistochemical data suggest that, in humans, orexins influence VTA DA and non-DA neurons at the somato-dendritic level, additional orexin-dopamine interactions may also take place at the level of DA terminals of VTA origin. Previous studies on rats revealed that some of the important mesolimbic terminal fields, including the central amygdala, the medial prefrontal cortex and the nucleus accumbens, receive abundant DA as well as orexinergic inputs [9]. Orexin receptor activation is excitatory in the amygdale [42] and the medial prefrontal cortex [43] and inhibitory [44] or excitatory [45] in the nucleus accumbens in different studies. It is important to recognize that orexinergic projections from the lateral hypothalamus may also regulate the DA systems of the VTA and the SN indirectly. Recent evidence suggests that GABAergic neurons of the tail of the VTA/rostromedial tegmental nucleus play a critically important role as a major GABAergic brake for VTA and SN DA systems [46]. Whether or not these GABAergic interneurons serve as additional targets for the descending orexinergic projections, requires clarification.

Coronal sections containing the VTA in our study also included the pars compacta of the SN. DA cells in the human SN tend to accumulate more neuromelanin pigment, in comparison with DA neurons of the VTA [47] which latter contain higher levels of vesicular monoamine transporter-2 for the vesicular packaging of dopamine and DOPA [48]. The analysis of orexinergic inputs to DA neurons of this region has shown that SN DA neurons of rats as well as humans receive direct innervation from hypothalamic orexinergic neurons. Although this input is quite sporadic, it is important to note that a similarly sparse innervation is capable of inducing robust behavioral effects in the rodent VTA [4], [29], [30], [40], [41]. Functional evidence also exists that orexins can act in the SN to influence important motor functions. Orexin A can significantly increase the time spent moving when injected into the pars compacta of the rat SN and D1 receptor activation is needed for the orexin A-induced increase in ambulation [49]. Interestingly however, DA neurons of the SN do not respond to orexins with excitation [19], unlike either VTA DA neurons [14], [36] or GABAergic interneurons in the pars reticulata of the SN [19].

In summary, in the present study we provide anatomical evidence for direct orexin B-IR inputs to subsets of DA and non-DA neurons of the human VTA and SN. Comparative analyses of sections from male rats and humans revealed that the innervation of VTA DA neurons is about three-five-times heavier in humans than in rats. We conclude that the role of orexin actions in the human VTA may be at least as important in reward processing and drug abuse, as established previously in rats.
